# Biomarkers for the Detection and Management of Hepatocellular Carcinoma in Patients Treated with Direct-Acting Antivirals

**DOI:** 10.3390/cancers14112700

**Published:** 2022-05-30

**Authors:** Loraine Kay D. Cabral, Luca Grisetti, Muhammad Yogi Pratama, Claudio Tiribelli, Devis Pascut

**Affiliations:** 1 Fondazione Italiana Fegato-ONLUS, AREA Science Park, Basovizza, 34149 Trieste, Italy; kay.cabral@fegato.it (L.K.D.C.); luca.grisetti@fegato.it (L.G.); yogi.pratama@fegato.it (M.Y.P.); ctliver@fegato.it (C.T.); 2 Doctoral School in Molecular Biomedicine, University of Trieste, 34127 Trieste, Italy

**Keywords:** hepatocellular carcinoma, HCV, biomarkers, DAA-treatment, serum, tissue

## Abstract

**Simple Summary:**

Chronic Hepatitis C virus (HCV) represents the main etiological factor for hepatocellular carcinoma (HCC) in developed countries. The introduction of direct-acting antivirals (DAAs) improved the eradication of the hepatitis C virus (HCV) but not the reduction in the incidence of HCV-associated HCC. Some patients still develop HCC, even after reaching a sustained virological response (SVR). This review is a summary of pre-clinical studies that investigated predictive biomarkers for HCC occurrence and recurrence in HCV-infected patients treated with DAAs. The presented biomarkers are found dysregulated in serum or tissue at specific time points (before, during, after DAA treatment or post SVR) and correlated with HCC-predisposing conditions. Thus, this review aims to improve the management of patients developing HCV-induced HCC.

**Abstract:**

Hepatocellular carcinoma (HCC) is the sixth-most common type of cancer worldwide and chronic Hepatitis C virus (HCV) represents the main etiological factor in developed countries. HCV promotes hepatocarcinogenesis through persistent liver inflammation and dysregulation of cell signaling pathways. The introduction of direct-acting antivirals (DAAs) resulted in a significant improvement in the eradication of the virus, with an expected reduction of HCC incidence. However, the risk of HCC development can persist after DAA treatment. Recent studies have investigated the potential use of molecular biomarkers that predict HCC occurrence or recurrence helping the stratification of patients under surveillance. This review aimed to summarize all pre-clinical exploration of predictive biomarkers to identify DAA-treated patients at risk for HCC development. Dysregulated microRNAs, lncRNAs, histone modifications, cytokines, proteins, and sphingolipids represent various classes of HCC risk predictors identified in two different biological sources (tissue and serum). The non-invasive serum markers can provide a more accessible means to perform clinical monitoring and predict the risk of HCC. In addition, conditions like cirrhosis, predisposing to HCC, strongly correlate with most of the molecular predictors identified, supporting the value of these molecules as possible biomarkers of HCC in DAA-treated patients.

## 1. Introduction

Primary liver cancer ranks sixth among the most common tumors in the world and it represents the third-most frequent cause of cancer-related death (905,000 new cases and 830,000 deaths in 2020) [1]. Worldwide, hepatocellular carcinoma (HCC) accounts for approximately 75% of the total liver cancers, being the most frequent liver malignancy [2]. The incidence of HCC is strongly correlated with the male sex and it increases progressively with advancing age [3]. Almost all cases of HCC are associated with a known etiology, most frequently chronic viral hepatitis (B and C), alcohol abuse, metabolic syndromes, and aflatoxin exposure [4]. Worldwide, approximately 31% of cases can be attributed to Hepatitis C virus (HCV) infection, which is considered the major risk factor for HCC in developed countries [3]. HCV is a single positive-stranded RNA virus [5] that can be a primary initiator of hepatic tumorigenesis through various direct and indirect mechanisms. Indeed, chronic hepatitis C infection leads to repetitive damage, regeneration, and inflammation, which often stimulates liver fibrosis, followed by the initiation of neoplastic clones accompanied by irreversible genetic and epigenetic alterations [6].

After the identification of HCV in 1989, HCV therapy became a central topic in research aiming to obtain viral eradication. The first discovered therapeutic agent was interferon (IFN), which later developed in pegylated IFN (PEG-IFN) [7]. IFN is a molecule generally released by host cells in response to the presence of several viruses [8], and it became the standard treatment for HCV patients, initially as a monotherapy cure, and subsequently in combination with ribavirin [7]. However, the success of IFN-treatment was defined by a partial early viral response (i.e., a ≥ 2log_10_ drop in HCV-RNA at week 12 of therapy), which offers a suboptimal predictive value for possible treatment [9].

The ability to manipulate the HCV genome as well as significant advances in the knowledge of HCV led to the development of the first direct-acting antivirals (DAAs), which strongly inhibit the replication of HCV by directly targeting essential viral proteins [7,10]. In 2011, the first DAAs were approved for clinical use, initially in the form of triple therapy in combination with PEG-IFN and ribavirin, but subsequently as IFN-free therapies since the combination of two or more DAAs improved efficacy and tolerability [10]. Moreover, the introduction of DAAs resulted in a significant improvement in the eradication of the virus [9]. The sustained virologic response (SVR) is reached when HCV RNA level is continuously undetectable for 12 (SVR12) or 24 (SVR24) weeks after the end of therapy [10], and it is associated with a lower risk of hepatic decompensation, coagulopathy, and ascites [11]. Until now, four main classes of DAAs have been developed that can target three different viral proteins: NS3/4A protease inhibitors, NS5A inhibitors, and two types of NS5B polymerase inhibitors (nucleoside and non-nucleoside inhibitors) [12]. DAA drugs approved by the European Medicines Agency (EMA) for the treatment of chronic HCV infections are reported in Table 1.

Despite the improvements obtained with the use of DAAs in the eradication of HCV, HCC may still develop in HCV-infected patients even after DAA-mediated SVR. A possible explanation was formulated by Robert in 2016 [13], suggesting that a reduction in the immune surveillance and/or the presence of small tumoral nodules still clinically undetectable before the initiation of DAAs might be responsible for the increased HCC risk in DAA-treated patients [13]. Indeed, DAA therapy can reduce the immune system response and thus promote a more rapid tumoral growth [14]. To date, the specific factors contributing to the risk of HCC in DAA-treated patients are still unclear. Hence, a complete understanding of the mechanisms that might drive and influence oncogenic progression after eradication of the infection is still warranted. In addition, markers that help the identification of individuals at high risk of HCC occurrence and recurrence after DAA treatment may result in interesting clinical tools to better stratify patients under surveillance.

In this review, we aimed to provide a better understanding of the HCC predictive biomarkers dysregulated in HCV-infected patients treated with DAA. The available evidence calls for the need to perform validation studies to determine the potential of these markers to predict HCC in a subset of patients, which can be further stratified based on the individual risk of developing *de novo* or recurrent HCC. Since HCC is a complex malignancy, which makes the identification of a single biomarker difficult, the panel of markers suggested in this review can be useful in detecting the early stages of the disease.

## 2. Molecular Mechanism of HCC Development in HCV Patients

**Figure 1:**
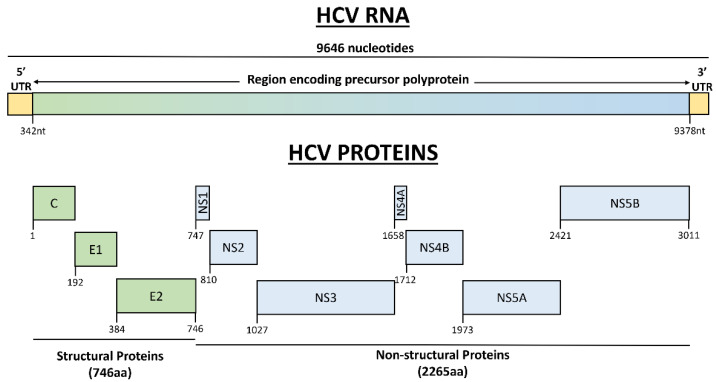
The organization of HCV genome and HCV transcribed proteins. The genome of HCV is composed of 9646 nucleotide bases. The central part of the genome is an open reading frame (ORF) of 9036 nucleotides flanked by 5′ (341nt) and 3′ (269nt) untranslated regions (UTRs). ORF encodes for a precursor polyprotein of 3011 nucleotides that is processed into 10 viral proteins. The three structural proteins are core protein (191aa) and two envelope glycoproteins (192aa and 363aa). The seven non-structural proteins show various lengths (NS1 63aa, NS2 217aa, NS3 631aa, NS4A 54aa, NS4B 261aa, NS5A 448aa, NS5B 591aa). UTR—untranslated region; C—core protein; E—envelope glycoprotein; NS—non-structural protein; nt—nucleotide; aa—amino acid.

HCV is an enveloped 9.6 kb single-positive-stranded RNA virus belonging to the *Hepacivirus* genus [15]. Until 2019, 8 major genotypes and 86 subtypes were confirmed, with possibly even more genotypes and subtypes to be identified (Figure 1) [16].

The first 6 HCV genotypes were identified before 2000, while genotypes 7 and 8 only in the last 8 years [16]. HCV genotype 7 was first identified in 2014 in the Democratic Republic of Congo and genotype 8 in 2018 in India [17,18]. Genotype 1 is the most prevalent worldwide, comprising 83.4 million cases (46.2% of all HCV cases), while genotype 5 represents less than 1% of total cases. Genotypes 4 and 5 are more frequent in lower-income countries, while genotypes 1 and 3 are widespread independently of the economic status of the country [19].

The HCV RNA is transcribed into structural proteins, including core protein and the envelope glycoproteins E1 and E2, and into non-structural proteins (NS1, NS2, NS3, NS4A, NS4B, NS5A, and NS5B proteins) (Figure 1) [15]. NS3-4A is a complex harboring protease and NTPase/ RNA helicase activities, NS5A is a phosphoprotein involved in the regulation of HCV RNA replication and interaction with host proteins, while NS5B is an RNA-dependent RNA polymerase [20]. All the various functions of the different proteins are summarized in Table 2.

The understanding of how HCV induces hepatocarcinogenesis has been extensively studied. Indeed, unlike HBV, whose integration of the viral genome in the host cell DNA leads to a direct oncogenic effect, the HCV genome is not directly integrated into the host genome [21,22]. However, as an oncogenic virus, HCV promotes the development of HCC in infected cells by several mechanisms consisting of (1) Persistent liver inflammation and immune-mediated oxidative stress damage due to chronic viral infection; (2) deregulation of cell signaling pathways such as cell cycle regulation, cell proliferation, and apoptosis, and (3) metabolic alterations, caused by viral proteins, leading to steatosis that further progress into fibrosis, cirrhosis, and HCC [23,24,25].

Chronic HCV infection stimulates the immune system activating persistent inflammation and inducing liver damage [26,27]. Chronic inflammation is induced by the accumulation of liver infiltrating lymphocytes in the liver [28]. Liver infiltrating T and B lymphocytes were observed to be higher in the cirrhotic parenchyma of HCV-infected patients with HCC *vs* patients without HCC [29]. Moreover, high levels of CD8^+^ T cells in cirrhotic nodules correlate to HCC occurrence and become a prognostic factor for recurrence after surgery [29]. Indeed, the increase in the CD8^+^ T cells and a decrease in the NK (Natural Killer) and NKT (Natural Killer T lineage) cells, which are involved in the immunosurveillance of cancer [30], are correlated with an evident dysregulation of the HCV infection and a consequent increase in the risk of HCC development.

In addition, chronic infection down-regulates the expression of liver-specific molecules such as hepatocyte nuclear factor 4 alpha (HNF4α) and miR-122, contributing to liver cirrhosis [31,32]. On the other hand, the persistent inflammation sustains the activation of signal transducer and activator of transcription 3 (STAT3), further promoting HCV replication and thus enhancing viral infection [32,33].

Genetic and epigenetic changes in host cells became relevant factors for hepatocarcinogenesis in HCV-infected patients. Indeed, dysregulated expression or activation of signaling mediators caused by mutations, chromosomal abnormalities, and epigenetic mechanisms contribute to liver carcinogenesis through the stimulation of erratic pathways [34,35]. Benegiamo et al. reported that HCV core protein upregulated DNA methyltransferase 1 (DNMT1) and 3b (DNMT3b), which in turn led to epigenetic alterations in liver cells [36]. Moreover, in vitro studies showed that infection of HCC cells with recombinant cell culture-derived hepatitis C virus resulted in suppression of histone H4 methylation/acetylation and histone H2AX phosphorylation, with a significant impact on the expression of essential genes for HCC development [37].

Viral proteins can directly or indirectly interact with several cellular key players that re-program host cells, promoting the expression of oncogenic genes [24,38]. Accumulating evidence showed that HCV core and non-structural proteins are involved in the activation of Wingless-related integration site (WNT)/β-catenin and Mitogen-activated protein kinase (MAPK) signaling pathway that paves the way for HCC development by altering cell cycle regulation, cell proliferation, inflammation, and cirrhosis progression [39,40].

HCV core protein can interact with the C-terminus of p53, increasing both DNA-binding affinity and transcriptional ability, which plays a crucial role in the regulation of the cell cycle and genomic integrity [41]. Core proteins can regulate the production of Transforming growth factor-β2 (TGF-β2) and Vascular endothelial growth factor (VEGF) proteins, which favor the development of hepatic angiogenesis in tumor development in patients with chronic HCV infection [42]. Furthermore, HCV core protein increases the production of reactive oxygen species (ROS), most probably through indirect mechanisms, and later impairs mitochondrial β-oxidation [43].

Envelope glycoproteins are also relevant in the activation of key processes for hepatocarcinogenesis. For example, the binding of E2 glycoprotein to C-type lectin immunoreceptors (CLRs) mimics the crosslinking of blood DC antigen 2 and DC-immunoreceptor. This mechanism inhibits the production of IFN-α and IFN-λ in plasmacytoid dendritic cells (pDCs), activating rapid phosphorylation of Ak strain transforming (AKT) and Extracellular Signal-Regulated Kinase 1/2 (ERK1/2) [44].

Not only structural but also non-structural proteins are involved in the re-programming of host cells activating the expression of oncogenes or downregulating the activity of tumor-suppressor genes involved in HCC development. NS3 can reduce the expression of Protein phosphatase Mg^2+^/Mn^2+^-dependent 1A (PPM1A) by promoting its ubiquitination and proteasomal degradation. The decrease in the levels of PPM1A promotes epithelial-to-mesenchymal transition (EMT), migration, and tumor invasion [45]. Einav et al. hypothesized that the guanosine triphosphatase activity encoded in the nucleotide-binding motif (NBM) of NS4B protein might play a key role in the transformation process, leading to tumor formation [46]. NS5A is involved in the mechanism for the downregulation of Growth arrest and DNA-damage-inducible-45α (GADD45α) via dysregulation of p53, which is in favor of cell proliferation [47].

Dysregulation induced by HCV involves also miRNAs [48]. In vitro studies showed that core and NS4B proteins can independently activate miR-27a and miR-27b expression, promoting lipogenesis and subsequently steatosis. MiR-27 targets include Angiopoietin-like 3 (ANGPTL3) and Peroxisome proliferator-activated receptor-α (PPAR-α) involved in the fatty acid β-oxidation, in the control of the cellular triglyceride content, and in the uptake from lipoproteins. Thus, the reduction of these genes appears to dysregulate the accumulation of triglycerides in vivo [49]. NS5A and NS3/A4 proteins can stimulate the binding of activator protein 1 (AP-1) on the miR-21 promoter, thus determining its up-regulation in infected cells. NS5A regulates miR-21 mainly through the Jun proto-oncogene (C-JUN), while NS3/4A through the Fos proto-oncogene (C-FOS). The miR-21 increase has been shown to target myeloid differentiation factor 88 (MyD88) and Interleukin-1 receptor-associated kinase 1 (IRAK1), which are involved in the production of HCV-induced type I IFN [50].

Pathological conditions such as hepatic steatosis and insulin resistance (IR) have been associated with chronic HCV infection and HCC development in infected patients [51]. Several studies have provided evidence on mechanisms that mediate the impairment of insulin signaling involving both Glucose transporter 2 (GLUT2) and Tumor necrosis factor-alpha (TNF-α). GLUT2, known to transport glucose to hepatocytes, is down-regulated by the HCV core protein, while TNF-α is upregulated during HCV infection [52]. Furthermore, TNF-α overexpression inhibits the Insulin receptor substrate (IRS) and Phosphatidyl-inositol 3 kinase via Suppressor of cytokine signaling 3 (SOCS3) [53], leading to a reduction in the uptake of glucose by cells [54]. Steatosis, on the other hand, represents a consequence of the derangement of lipid metabolism caused directly by HCV [55]. HCV proteins induce the accumulation of lipid droplets as well as lipogenic gene expression and protein activity [56]. Peroxisome Proliferator-Activated Receptor Alpha/Gamma (PPAR-α/γ) is strongly involved in lipid and lipoprotein metabolism and seems to have a protective effect against hepatic inflammation and fibrosis and it is reduced by the activity of the HCV core protein [57]. In addition, the sterol regulatory element-binding protein-1c (SREBP-1c), a transcription factor regulating lipogenesis, is activated by the HCV core protein, leading to hepatic fat accumulation [58]. These conditions can be worsened by the concomitant HCV-induced or host-related IR [54].

All these studies appear to highlight the relevant role of HCV in HCC development, as summarized in Table 3. Indeed, chronic HCV infection stimulates the immune system to develop liver inflammation and in turn, the persistent liver inflammation enhances HCV replication. In addition, the HCV structural and non-structural proteins are reported to be able to target cell signaling pathways that re-program host cells promoting the expression of oncogenes and downregulating tumor-suppressor genes, which leads to the development of HCC-predisposing conditions.

## 3. The Controversial Role of DAA Treatment in HCC Development

The emergence of DAAs has not only significantly improved treatment for HCV but also reduced the incidence of HCC. Indeed, patients who achieve SVR with DAA treatments have been shown to have improved life expectancy and reduced incidence of HCC compared with untreated patients and those with a failed treatment [60]. HCV infection stimulates the TGF-β pathway inducing cell plasticity in liver fibrosis, a condition that can be exploited by cells to promote hepatocarcinogenesis [61,62]. Therefore, significantly lower TGF-β1 serum levels were identified in chronic HCV patients who achieve DAA-mediated SVR, suggesting that down-regulation of TGF-β is correlated with a reduction of viral replication and an increase of sensitivity to treatment [63]. Moreover, DAAs impact the risk of HCC by reducing the levels of miR-122, involved in HCV replication, and promoting the restoration of innate immunity and down-regulation of INF-II and INF-III, associated receptors, and target genes [64].

To date, conflicting results on the efficacy of DAAs in reducing the risk of HCC development have been reported in the literature. Wang et al. reported the absence of differences in the expression of several growth factors responsible for HCC development, such as VEGF and Platelet-derived growth factor (PDGF), between patients that do or do not achieve SVR [65]. Moreover, the possibility to develop post-SVR HCC seems to be higher in patients who receive DAAs compared with IFN therapy [64,66]. An explanation lies in the fact that patients receiving DAA therapy are generally older and with higher fibrosis stages, resulting in a higher overall incidence of HCC, regardless of treatment [67].

However, increasing evidence suggests that patients who achieve SVR with DAA therapy do not have a significantly greater HCC risk than those treated with IFN [67]. A hypothesis holds that malignancy occurrence could be influenced by persisting pro-oncogenic environment and reduced immune response after HCV clearance [68]. The level of pro-inflammatory immune factors such as interleukin-4 (IL-4) and interleukin-13 (IL-13) was found higher before, during, and after DAA therapy in patients developing HCC, strengthening the existence of a specific immune mechanism contributing to HCC development, pre-existing and persisting after SVR [69]. Thus, predictive markers that identify individuals with a higher risk to develop HCC in HCV-induced pathologies, even after DAA treatment, are still needed.

## 4. Predictive Biomarkers of HCC Development in DAA-Treated Patients

The risk of HCC development can persist after HCV treatments, despite many efforts that have been already invested in the development of new HCV cures. However, Ono et al. suggested that HCC risk can be monitored by liver transcriptome signatures [70]. This underlines the advantage of the use of specific biomarkers to predict HCC in HCV-infected patients treated with DAAs [71]. Various biomolecules have been observed as dysregulated before, during, or after DAA treatment, being potentially attractive as biomarkers. Thus, new predictive tools, alongside the usual surveillance for HCV-infected patients, can improve the identification of patients at higher risk of HCC occurrence and recurrence.

In an attempt to consolidate published information to manage DAA-treated patients, we investigated several reports presenting potential markers that can predict the development of malignancy. These biomarkers are grouped based on the biological sources of origin: (A) Tissue biomarkers or (B) serum biomarkers to predict the risk of HCC in DAA-treated patients (Table 4). Moreover, we reported the specific time point at which these molecules can be used to predict HCC development (before, during, or after DAA therapy, or post SVR, Table 4). In addition, we grouped biomarkers that can be used to identify patients at risk for *de novo* and/or recurrent HCC (Table 4).

### 4.1. Tissue Biomarkers to Predict the Risk of HCC in DAA Treated Patients

Accumulating evidence reported that about 20% of HCV-infected individuals develop cirrhosis in 20–30 years [72,73], and 1%–4% develop HCC [72]. Since the progression to cirrhosis and lately to HCC is associated with molecular alterations, several studies were able to identify specific markers in liver tissue of patients who later developed HCC. As a consequence, the monitoring of such dysregulations may be of precious help in determining patients at risk of malignancy [74,75,76].

#### 4.1.1. Angiogenic Factors

Villani et al. speculated that the increased risk of tumor recurrence in DAA-treated patients might derive from a drug-induced angiogenic mechanism [77]. Published reports showed evidence of elevated levels of circulating VEGF during [77] and after [75] DAA treatment. VEGF is known to contribute to the induction of Angiopoietin 2 (ANGPT2), a growth factor belonging to the angiopoietin/Tie (tyrosine kinase with Ig and EGF homology domains) signaling pathway, which regulates endothelial permeability and angiogenic functions [78]. According to the work of Kunz et al., in the presence of advanced liver fibrosis and reduced blood flow, an increased level of VEGF activates ANGPT2, subsequently leading to the formation of new vasculature [79].

In the study of Faillaci et al. ANGPT2 expression was observed to be elevated in cirrhotic tissue and primary tumor liver tissues of susceptible patients with activated neoangiogenesis (those with severe fibrosis and splanchnic collateralization) [75]. The high levels of ANGPT2 before DAAs were independently related to the risk of HCC recurrence (odds ratio (OR), 1.137; 95% confidence interval (CI), 1.044–1.137; *p* = 0.003) and occurrence (OR, 1.604; 95% CI, 1.080–2.382; *p* = 0.019), thus suggesting the possible role of ANGPT2 as a biomarker for identifying patients with cirrhosis at risk of HCC development before the treatment with DAAs (Table 4).

#### 4.1.2. Epigenetic Footprints

Histone modifications play a fundamental role in gene regulation through the dynamic remodeling of chromatin [80]. In such modifications, viruses can be involved and subsequently affect transcription in host cells [81]. There is evidence of epigenetic signatures related to HCV infection observed both in vitro models [74] and in human samples [76]. These signatures remain persistent even after viral eradication [74,76].

By comparing 8 patients with chronic HCV infection, 21 patients with DAA- or IFN-based curative therapy, and 6 non-infected controls, Hamdane et al. investigated epigenetic alterations characterizing HCV infection [76]. Particularly, they identified genome-wide changes in the acetylation of the lysine residue on the histone H3 (H3K27ac) correlating with changes in the expression of various mRNAs and proteins [76] associated with liver fibrosis. Interestingly they observed that H3K27ac changes persisted in the F4 stage (advanced fibrosis/cirrhosis), in which the HCC risk is higher, while they were reduced in fibrosis stage F2–F3 [76].

Some of the genes associated with H3K27ac changes in liver tissue belonged to oncogenic pathways which include angiogenesis and cell proliferation [76]. Alterations induced by H3K27ac changes involve the oncogene Sphingosine kinase 1 (SPHK1), a major regulator of cell apoptosis inhibition and proliferation promotion [82]. Hamdane et al. reported an upregulation of SPHK1 protein levels during HCV infection that remained elevated after DAA treatment [76]. They also investigated SPHK1 on paired liver tissues (HCC and adjacent non-tumorous tissues), observing an increased expression in non-tumorous adjacent tissues. Their evidence could indicate the presence of changes before oncogenesis and even after viral cure [76]. By reconsidering previously published data, Hamdane et al. were able to observe a strong correlation of high SPHK1 expression with HCC risk in patients with HVC-induced cirrhosis, even in subjects reaching the SVR, although treated with IFN-based therapies. In addition, a positive association between SPHK1 expression and tumor size, tumor stage, and histological differentiation was reported [83].

Hence, persistent H3K27ac modifications, observed especially in advanced liver disease (F4 fibrosis/cirrhosis), coupled with an elevated SPHK1 expression can be a good HCC predictor in patients reaching SVR.

On the other hand, acetylation on lysine 9 of histone H3 (H3K9ac) was observed by Perez et al. in HCV-infected patients [74]. These alterations were observed mainly in the correspondence of eight genes (WNT Family Member 10A (WNT10A), JunB proto-oncogene (JUNB), FOS-like 2 (FOSL2), MYCN proto-oncogene (MYCN), TNF-α induced protein 3 (TNFAIP3), Kruppel-like factor 4 (KLF4), Endothelin 1 (EDN1), and Proprotein convertase subtilisin/Kexin type 9 (PCSK9)) of seven HCV-infected patients and four DAA-treated patients reaching SVR, indicating that, despite the viral clearance, those epigenetic changes persisted in patients. Additionally, an increased risk of HCC occurrence after 2.5 years was reported to be associated with dysregulated expression of those eight genes, highlighting that the risk of HCC slowly increases in correspondence with HCV-induced epigenetic alteration [74]. Other reports evaluated the eight-gene signature in liver biopsies from 216 patients with HCV-related Child-Pugh class A cirrhosis prospectively followed up for a median of 10 years and demonstrated a significant correlation between HCC development in HCV-infected patients and altered levels of the gene signature (hazard ratio (HR) = 3.18, *p* = 7.8 × 10^−5^) [84]. 

Further investigations focused on confirming the role of these biomarkers in predicting HCC occurrence in DAA-treated patients would be a significant step toward the use of such markers in a clinical setting. These studies underline the significance of epigenetic changes, such as H3K9ac (post SVR) and H3K27ac (during, after DAA, and post SVR) in the identification of DAA-treated patients with a higher risk of HCC (Table 4).

### 4.2. Serum Biomarkers to Predict the Risk of HCC in DAA-Treated Patients

Though ANGPT2 and epigenetic factors hold a promise for possible use in patients’ stratification and follow-up, it should be considered that they are tissue biomarkers, and a biopsy is required to assess patients’ risk. Thus, biofluids appear more attractive for the search for biomarkers in predicting HCC occurrence or recurrence.

#### 4.2.1. Alpha-Fetoprotein

Serum alpha-fetoprotein (AFP) is still the most common serum biomarker for screening and diagnosis of HCC. However, it does not solely indicate the presence of HCC since elevated AFP levels are also found in patients with hepatitis infection and cirrhosis only [85]. Though AFP performances are not generally satisfactory, Yoshimasu et al. identified AFP and AFP-L3% of possible interest as circulating biomarkers to assess the risk of HCC occurrence or recurrence in DAA-treated patients [86].

Particularly, levels of AFP were increased in HCV patients with occurrence or recurrence of HCC, both before and after the DAA treatment, whereas the expression of AFP-L3% was associated with the occurrence and recurrence of HCC only after DAA therapy [86]. Other studies supported the role of AFP measured at the end of DAA therapy as a predictor of HCC development [87,88] while, to our knowledge, only one study showed the value of pre-DAA treatment AFP levels as predictors of HCC recurrence [89] and no association between the AFP-L3% and incidence of HCC after DAAs has been reported yet.

#### 4.2.2. WFA^+^-M2BP

The serum level of Wisteria floribunda agglutinin positive Mac-2-binding protein (WFA^+^-M2BP) was identified as a predictive biomarker for HCC occurrence after DAA therapy in patients with fibrosis but without previous history of HCC [87]. WFA^+^-M2BP is secreted by hepatic stellate cells (HSCs) and induces the expression of Mac-2 in Kuppfer cells, determining the production of extracellular matrix by HSCs, contributing to the creation of a supportive microenvironment for HCC growth [90]. Patients with subsequent HCC development had a higher level of serum WFA^+^-M2BP after the DAA treatment compared with the ones who did not develop HCC (Cut-off index (COI): 2.86 ± 2.2 vs. 1.62 ± 1.6). Patients with higher levels of WFA^+^-M2BP (COI ≥ 1.75) at 12 weeks after SVR presented more than five-times higher episodes of HCC development, during the one-year follow-up (5.3% vs. 0.9%). Thus, WFA^+^-M2BP resulted as a factor significantly associated with the development of HCC (HR = 6.0 (95% CI, 1.8–19.4) in the studied population. Additionally, the authors were able to observe that the levels of WFA^+^-M2BP can reflect the fibrosis grade after SVR. In a stratified analysis, the usefulness of this biomarker was significantly observed only in patients with advanced liver fibrosis (F3-F4)/cirrhosis but not in early fibrosis grade (F0–F2) [87]. Indeed, the incidence of HCC development was considerably low in F0–F2 subjects, especially in a short-term follow-up. Hence, WFA^+^-M2BP was suggested as a marker associated with the background liver environment such as fibrosis. On the other hand, for the patients with a history of HCC, the determined value for WFA^+^-M2BP levels was found higher (COI ≥ 3.0) vs the value in *de novo* HCC patients (COI ≥ 1.75) in identifying the risk of recurrence. However, it is important to note that post-SVR AFP of ≥ 6 ng/mL was the only independent factor associated with the risk of recurrence (HR = 3.1 (95% CI, 1.3–7.5). The authors speculated that for patients with prior history of HCC, WFA+-M2BP has a low predictive value in assessing the risk of recurrence [87].

#### 4.2.3. Circulating Immune Mediators

Chronic HCV infection stimulates the immune system, activating persistent inflammation and inducing liver damages [26,27]. Indeed, it is expected that the perturbations induced by the chronic infection may persist even after the SVR, despite the curative role of DAA treatment in eradicating HCV and possibly lowering the risk of developing HCC [91]. Therefore, some immune changes were hypothesized to favor the progression of pre-malignant lesions present before the DAA treatment [92]. Several studies investigated the role of various immune mediators in the possible identification of patients at risk for HCC development after reaching the SVR.

Both works of Debes et al. and Jilkova et al. observed that specific immune mediators were already increased before the start of DAA administration in patients developing HCC after SVR [69,93]. Thus, with the intent to identify a specific immune profile predicting hepatocarcinogenesis in DAA-treated patients with severe liver fibrosis/cirrhosis (F3-F4), Debes et al. analyzed twelve cytokines, growth factors, and apoptosis markers before DAA treatment, observing that nine of them had increased levels in the serum of patients developing HCC after DAA treatment [93]. Monokine induced by gamma interferon (MIG), interleukin 22 (IL-22), and tumor necrosis factor (TNF)-related apoptosis-inducing ligand (TRAIL) showed an AUC value above 0.80, and A proliferation-inducing ligand (APRIL), VEGF, IL-3, the cytokine TNF-related weak inducer of apoptosis (TWEAK), stem cell factor (SCF), and IL-21 even above 0.9, in discriminating patients developing *de novo* HCC after DAA treatment. Authors hypothesize that these differences in the levels of circulating immune mediators may derive from an ongoing oncogenic or pre-oncogenic activity brought by the damages of the HCV infection [93]. Among the eight markers, TRAIL, VEGF, and IL-22 were already reported to be associated with HCC even in different etiologies [94].

In addition to this panel of potential predictors of HCC in DAA-treated patients, IL-4 and IL-13 were found to be significantly increased before DAA treatment in patients who later developed HCC [69]. The two markers were consistently high in the serum of HCV patients at the start, during, and at the end of therapy, even 3 months after the end of treatment. IL-4 and IL-13 are generally associated with carcinogenesis and play a crucial role in immune-related mechanisms [69], emphasizing that the dysregulation of immune mechanisms caused by the HCV infection is not reversed by DAA treatment and can still drive progression to malignancy.

#### 4.2.4. Non-Coding RNAs

Non-coding RNAs (ncRNAs) are regulatory elements involved in various cellular mechanisms that favor liver homeostasis among other functions [95]. Since their discovery, they paved the way for new studies exploring their role as disease biomarkers. Of particular importance are the circulating ncRNAs that can be measured in biofluids, making them an easily accessible source of disease-related information. The advantage of the use of serum ncRNAs as potential biomarkers is highlighted by their stability in biofluids and detectability through non-invasive methods [96,97].

El-Khazragy et al. were the first to analyze long non-coding (lnc) RNAs as potential biomarkers of HCC risk after DAA treatment [98]. In a cohort of 220 HCV genotype 4-infected patients with compensated cirrhosis, 17% developed HCC (HCC+) over the 12 months following the therapy. The HCC+ patients presented significantly higher levels of lnc-HOTAIR in serum (68-fold changes (FC) vs 24 FC), compared with controls (HCC–). The overexpression of lnc-HOTAIR was correlated with an increased level of viral copies, high level of AFP (>200 IU/mL), presence of lymph nodal infiltration, extrahepatic metastasis, and large tumor size (>5 cm) [98]. In addition, according to previous studies, lnc-HOTAIR was found to be higher in HCC tissues in comparison with paired non-cancerous ones [99,100], suggesting its potential as a marker for malignancy. Moreover, lnc-HOTAIR showed the potential to be a predictive biomarker for HCC before and during DAA therapy in chronic HCV genotype 4 patients with a sensitivity and specificity of 84% and 82%, respectively [98].

Evidence about the predictive role of miRNAs in the development of HCC particularly in DAA-treated patients was provided by Pascut et al. [101]. The study analyzed serum miRNA levels of cirrhotic patients who developed HCC following DAA treatment (HCC+) and patients who did not develop HCC (HCC–). The expression of miR-3197 was downregulated in the serum of HCC+ patients, both before and one month after DAA treatment, making miR-3197 a biomarker candidate to predict the development of HCC in HCV patients with a sensitivity of 80% and 86% and specificity of 80% and 73%, respectively (before treatment: Area under the curve (AUC) = 0.78 (95% CI, 0.53–0.90); after treatment: AUC = 0.80 (95% CI, 0.52–0.92) [101]. In addition, miR-3197 was downregulated in the liver of patients having a previous history of HCC and with episodes of tumor recurrence, thus supporting the role of this miRNA as a possible biomarker for both HCC occurrence and recurrence [102].

Itami-Matsumoto et al. reported dysregulated levels of numerous serum exosomal miRNAs in DAA-treated patients with subsequent HCC development at SVR12 [103]. In a cohort of 41 liver cirrhotic patients with a prior history of HCC, 16 (39%) developed HCC. Three miRNAs (miR-4718, miR-6511a-5p, and miR-642a-5p) were able to predict HCC recurrence with accuracy and specificity of 88.5% and 87.8%, respectively. Moreover, analyzing a cohort composed of 69 among cirrhotic and non-cirrhotic subjects with prior cured HCC, a panel composed of four miRNAs (miR-211-3p, miR-6826-3p, miR-1236-3p, and miR-4448) discriminated the 25 patients with HCC recurrence from the controls with accuracy and specificity of 85.3% and 85.3%, respectively. In addition, they were able to identify two relevant miRNAs (miR-762 and miR-8069) for the discrimination of patients (15 subjects) with HCC occurrence in a cohort of 70 individuals with or without cirrhosis (accuracy = 83.3%; specificity = 87.1%). Among the nine dysregulated miRNAs, the serum levels of miR-4718, miR-642a-5p, miR-6826-3p, and miR-762 were positively correlated with the expression in liver tissue, and further in vitro investigation showed their involvement in cell proliferation and apoptosis [103].

#### 4.2.5. Sphingolipids

A panel of serum sphingolipids (C16Cer, C24DHC, and C24:1DHC) has been used to predict the HCC risk in patients with liver cirrhosis after therapy. In their study, Mücke et al. profiled the serum sphingolipids in 166 patients with HCV-cirrhosis and SVR both at baseline and 12 weeks after completion of DAA therapy [104]. Interestingly, in cirrhotic patients with HCC occurrence, serum C24DHC, C24:1DHC, and C16Cer were significantly increased at SVR12. These sphingolipids were able to discriminate patients with a future occurrence of HCC with an AUC above 0.7 (C16Cer: AUC = 0.741 (95% CI, 0.573–0.908), C24DHC: AUC = 0.746 (95% CI, 0.565–0.928), C24:1DHC: AUC = 0.730 (95% CI, 0.563–0.897). In addition, C16Cer was able to predict the risk of HCC with high diagnostic accuracy, even in AFP-negative patients (AUC = 0.766, OR = 1.030 (95% CI, 1.005–1.056)), highlighting a possible superior value of this biomarker compared with AFP [104]. Therefore, Mücke et al. contributed to the identification of another class of non-invasive biomarkers that can be helpful in the surveillance of those patients at risk of HCC.

## 5. Validation of Pre-Clinical Biomarkers

DAAs offer the best treatment for HCV-infected patients. However, despite the efficiency to eradicate the virus, the risk of tumor development is still not eliminated. Until now, AFP is the only serum biomarker that has undergone pre-clinical validation [105]. However, it offers only modest sensitivity and specificity for early diagnosis of HCC [106].

To our knowledge, the predictive biomarkers in this review have proven their potential in determining the risk of HCC; however, all these markers are still in the exploratory stages and require extensive validation steps including retrospective longitudinal studies, prospective screening, and lastly cancer-control studies before becoming a useful tool in the clinical practice [105]. Several challenges in biomarker studies including incomplete cohort data, selection bias, and limited sample size might reduce the potential of the suggested biomarkers, and hence further accurate evaluations of the studies should be performed to select the best candidates for subsequent validation phases [107].

Since the current management of DAA-treated patients still lacks the tools to determine individuals at risk of developing HCC, it is necessary to identify a reliable panel of markers that will answer this need.

Since HCC is a complex malignancy, a panel of markers can better picture patients’ risk. In addition, non-invasive serum markers are particularly appreciable for longitudinal monitoring to better stratify patients and finally improve the quality of healthcare.

**Table 4:** Biomarkers in predicting HCC occurrence and recurrence in DAA-treated patients.

Source	Biomarkers	Dysregulation in Relation to DAA Treatment				Risk Predictor of HCC		Cohort Size	Refs.
		Before	During	After	Post SVR	Occurrence	Recurrence		
Tissue Biomarkers	ANGPT2	↑				√	√	242	[75]
	H3K27ac + SPHK1 H3K27ac (genome-wide changes) SPHK1					√	--	48	[76]
			↑	↑	↑				
	H3K9ac + Panel of 8 genes H3K9ac (dysregulated) WNT10A, JUNB, FOSL2, MYCN TNFAIP3, KLF4, and EDN1 PCSK9					√	--	17	[74]
					↑				
					↓				
Serum Biomarkers	AFP	↑		↑		√	√	234	[86]
	AFP-L3%			↑		√	√	220	[86]
	lnc-HOTAIR (in HCV genotype 4 infected patients)	↑	↑			√	--	23	[98]
	Panel of circulating immune mediators MIG, IL-22, TRAIL, APRIL, VEGF, IL-3, TWEAK, SCF, IL-21, IL-4 and IL-13	↑	↑	↑		√	--	49	[69,93]
	miR-3197	↓		↓		√	--	60	[101]
	Panel of Sphingolipids C16Cer, C24DHC, and C24:1DHC				↑	√	--	166	[104]
	WFA+-M2BP			↑	↑	√	--	567	[87]
	Panel of exosomal miRNAs miR-4718, miR-6511a-5p, and miR-642a-5p miR-211-3p, miR-6826-3p, miR-1236-3p, and miR-4448				↓	--	√	139	[103]
	miR-762 and miR-8069				↓	√	--	139	[103]

↑—upregulated ↓—downregulated √—predictive of (occurrence/recurrence).

## 6. Future Perspectives

Insights into characterizing and discriminating HCV-induced HCC patients treated with DAAs may add new perspectives to post-SVR management. This could allow the use of therapeutic agents targeting specifically HCV-associated molecular aberrations and improve management of HCV-induced HCC, possibly alleviating the burden of HCC mortality and morbidity.

To date, the available information about these predictive biomarkers is still very limited. Therefore, further analyses and validations of the potentiality of the markers presented in this review can eventually offer better possibilities for surveillance in DAA-treated patients. New studies based on larger cohorts that can effectively discriminate the development or not of HCC in DAA-treated patients are still warranted.

## 7. Conclusions

HCC development in DAA-treated patients has raised significant concerns in clinical settings. Treatment and management for chronic HCV infection have improved over the years and the use of DAA therapy contributed to successful viral eradication. Hence, the promising effects of these treatments were expected to greatly reduce the risk for liver malignancy. However, accounts of HCC occurrence and recurrence have been observed in HCV-infected patients despite the use of DAA treatments and the achievement of SVR. This could be a result of the activation of pro-oncogenic pathways during the long and chronic exposure to the virus, a reduction of the immunosurveillance during DAA therapies, and the presence of previously undetectable lesions which grow rapidly during DAAs. Specifically, pre-oncogenic conditions like severe inflammation, advanced fibrosis, cirrhosis, and the presence of malignancy before DAA treatment are identified as risk factors increasing the chances of HCC despite SVR. Thus, it is noteworthy to discuss that, while DAA treatment can clear the viral infection, it does not reverse physiological damages induced by the virus.

Hence, this review emphasized different biomarkers associated with HCV infection and HCC predisposing conditions, as summarized in Table 4. Most molecules are associated with HCV-induced damages that eventually promote cancer and are persistently dysregulated during, after DAA therapy, and post SVR. Interestingly, some altered expression levels are already detectable before the initiation of DAA therapy, thus providing a molecular tool to stratify patients at risk, even before DAA therapy initiation. The availability of different biological sources (tissue and serum) allows the possibility to access the condition and risk of DAA-treated patients more comprehensively. Moreover, the use of non-invasive sources such as serum is more convenient for patient surveillance.

Two important aspects of HCV-associated HCC after DAA treatment should be considered in light of the improved patients’ stratification: (1) the presence of pro-oncogenic conditions (fibrosis and cirrhosis), and (2) the persisting dysregulation of biological markers before, during, and after DAA treatment. Taken together, these two factors may provide the basis for differential surveillance and management for patients at risk.

## Figures and Tables

**Table 1:** Direct-acting antivirals approved by EMA for the treatment of chronic HCV infection.

DAAs	Components	Targets
Daklinza ^1^	Daclatasvir	NS5A
Epclusa	Sofosbuvir	NS5B
	Velpatasvir	NS5A
Exviera	Dasabuvir	NS5B
Harvoni	Lepidasvir	NS5A
	Sofosbuvir	NS5B
Maviret	Glecaprevir	NS3/NS4A
	Pibrentasvir	NS5A
Olysio ^1^	Simeprevir	NS3/NS4A
Sovaldi	Sofosbuvir	NS5B
Viekirax	Ombitasvir	NS5A
	Paritaprevir	NS3/NS4A
	Ritonavir	NS3/NS4A
Vosevi	Sofosbuvir	NS5B
	Velpatasvir	NS5A
	Voxilaprevir	NS3/NS4A
Zepatier	Elbasvir	NS5A
	Grazoprevir	NS3/NS4A

^1^ The drugs are not authorized anymore by EMA.

**Table 2:** Functions of HCV proteins.

Gene	Proteins	Functions
Structural proteins		
Core (C)	p22	Nucleocapsid
E1	gp35	Envelope glycoprotein
E2	gp70	Envelope glycoprotein
Non-structural proteins		
NS1	p7	Short membrane peptide with possible ion channel function
NS2	p23	Cysteine protease
NS3	p70	Serine protease, RNA helicase with NTPase activity
NS4A	p8	Cofactor for NS3
NS4B	p27	Integral protein inducing membranous web formation
NS5A	p56/p58	Poly-phosphoprotein involved in HCV replication, modulation of cell signaling pathways, and mediation of IFN response
NS5B	p68	RNA-dependent RNA polymerase

**Table 3:** HCV proteins contribute to the development of malignancy by modulating cellular gene expression and pathways.

Pathway	Gene	HCV Protein	Reference
Angiogenesis	TGF-β2, VEGF	Core	[42]
Cell cycle regulation	--	NS5A	[59]
Cell cycle regulation and DNA repair	p53	Core	[41]
Cell cycle regulation and proliferation	MAPK/ERK pathway members	Core	[40]
EMT and invasion	PPM1A (ubiquitination)	NS3	[45]
Epigenetic changes	DNMT1/DNMT3	Core	[36]
IFN reduction	C-JUN, C-FOS, AP-1, miR-21, MyD88, IRAK1	NS5ANS3/NS4A	[50]
Inflammation and cirrhosis progression	WNT/β-catenin pathway members	Core and NS5A	[39]
Immune escape	CLR, INFα/γ, Akt, ERK1/2	E2	[44]
Lipid accumulation	SREBP-1c	Core	[58]
Lipid metabolism and inflammation	PPARα	Core	[57]
Lipid metabolism and hepatic steatosis	miR-27a/27b, ANGPTL3, PPARα	NS4B	[49]
Oxidative stress	--	Core	[43]
Proliferation	GADD45α	NS5A	[47]
